# Morphological and organic spectroscopic studies of a 44-million-year-old leaf beetle (Coleoptera: Chrysomelidae) in amber with endogenous remains of chitin

**DOI:** 10.1038/s41598-023-32557-w

**Published:** 2023-04-11

**Authors:** Jerit L. Mitchell, Ryan C. McKellar, Mauricio Barbi, Ian M. Coulson, Andris Bukejs

**Affiliations:** 1 Department of Physics, University of Regina, Regina, SK S4S 0A2 Canada; 2 Royal Saskatchewan Museum, 2445 Albert St., Regina, SK S4P 4W7 Canada; 3 Department of Biology, University of Regina, Regina, SK S4S 0A2 Canada; 4 Department of Ecology & Evolutionary Biology, University of Kansas, Lawrence, KS 66045 USA; 5 Department of Geology, University of Regina, Regina, SK S4S 0A2 Canada; 6 Institute of Life Sciences and Technologies, Daugavpils University, Vienîbas 13, Daugavpils, 5401 Latvia

**Keywords:** Palaeontology, Entomology, Scanning electron microscopy, Infrared spectroscopy, X-ray tomography

## Abstract

This study details the quality of preservation of amber deposits in the Eocene. Through Baltic amber crack-out studies using Synchrotron Micro-Computed Tomography and Scanning Electron Microscopy it was found that the cuticle of a specimen of leaf beetle (*Crepidodera tertiotertiaria* (Alticini: Galerucinae: Chrysomelidae)) is exceptionally well preserved. Spectroscopic analysis using Synchrotron Fourier Transform Infrared Spectroscopy suggests presence of degraded $\upalpha$-chitin in multiple areas of the cuticle, and Energy Dispersive Spectroscopy supports the presence of organic preservation. This remarkable preservation is likely the result of several factors such as the favourable antimicrobial and physical shielding properties of Baltic amber as compared to other depositional media, coupled to rapid dehydration of the beetle early in its taphonomic process. We provide evidence that crack-out studies of amber inclusions, although inherently destructive of fossils, are an underutilised method for probing exceptional preservation in deep time.

## Introduction

Amber (fossilized plant resin) inclusions are a crucial source of information for reconstruction of ancient ecosystems as they are biased towards smaller animals, such as insects, that are otherwise not well represented in sedimentary settings^1^. These inclusions often feature “life-like” 3D preservation, providing significantly more morphological information than cast or compression fossils^2^. This leads to common preservation of cuticle^3^ (including structural colours^4,5^), as well as internal soft tissue structures of invertebrates^2^ in amber. In larger pieces of amber, high fidelity vertebrate preservation can be found in smaller-bodied specimens^6,7^ or parts of specimens such as feathers^8^. Plants^3^, fungi^9^, and microbes^10^ are also well represented in amber. Attempts have been made to determine organic contents inside amber inclusions using non-invasive methods^11–13^, however, they are limited in their resolution and scope to provide molecular identification.

Despite the large volume of reports on exceptional morphological preservation, there are very few modern studies attempting to extract organics from inside amber. The shielding and anti-biotic properties of amber provides the potential to preserve organic molecules better than any other medium^14^. Generally, fossil materials such as bones are porous, leaving them exposed to interaction with surrounding sediment and pore fluids. If there are organic components left in fossils, they will generally be outnumbered by inorganic material in the surrounding tissues or host rocks. The effects of weathering and diagenesis, along with possibility of exogenous organic material as contaminants make spectral interpretation more difficult^15^. Fortunately, amber as a preservational medium provides an essentially closed system (except for exceptional circumstances^16^), so that inorganic and organic contributions from sediment are expected to be minimal. Exposing amber inclusions through a crack-out study of well-preserved specimens provides an opportunity to test for organic material under optimal preservation conditions.

The organic molecule that has the greatest potential to be preserved in insects is chitin from their exoskeletons^17^. Chitin $(\text{C}_8 \text{H}_{13} \text{O}_5 \text{N})_n$ is a glucose based structural aminopolysaccharide that is abundant in nature, found in the skeletal structures of many invertebrates such as sponges^18^, corals^19^, crustaceans^20^, arachnids^21^, cell walls of fungi^22^, and the cuticles of insects^23^. There are three known polymorphs of chitin: $\upalpha$-chitin found in arthropods, fungi and sponges; $\upbeta$-chitin found in mollusks and diatoms; and a rare $\upgamma$-chitin form found in insect cocoons^24,25^. For insects in particular, chitin helps strengthen the exoskeleton as part of the protein-chitin complex in the cuticle, and is expected to decay less readily compared to other organic macro molecules such as DNA or proteins^17^. However, chitin is not commonly found in the fossil record past one million years^15^. The oldest accepted fossil insect chitin ($\scriptstyle \sim$25 Ma) was a beetle found in lacustrine shale from the Enspel Lagerstätte, Germany^26^, described in 1997 . Since then, there have been few chitin preservation claims in the literature for time periods after the Oligocene for any animal (claims include: $\scriptstyle \sim$34 Ma cuttlefish^27^, $\scriptstyle \sim$200 Ma gastropod egg capsule^28^, chitin-protein complex in $\scriptstyle \sim$310 Ma scorpion and $\scriptstyle \sim$417 Ma eurypterid^29^, $\scriptstyle \sim$505 Ma sponge^30^, 810 to 715 Ma fungi micro fossils^31^). While these studies featured fossils from shale settings, even fewer chitin claims are from resins. One study^11^ was able to use X-ray Raman scattering to find evidence of polysaccharides similar to chitin in the cuticle of an ant from Eocene amber.

Baltic amber is found around the Baltic Sea region and dates back to the Eocene epoch. The deposit formed $\scriptstyle \sim$44 Ma, and is known for its well-preserved insect inclusions^14^. Preservation in amber is best when there is rapid dehydration of the insect upon death, allowing the insect’s cuticle to detach from the amber matrix^2^. In these cases chitinous fibres and even original cuticle colour can be seen^4^. Baltic amber is rich in biodiversity including the genus *Crepidodera* that is known to contain over 40 modern species, and is part of a much larger family of leaf beetles (Chrysomelidae)^32^. There are currently three known fossil species of *Crepidodera*: *C. decolorata*^33^, *C. svetlanae*^34^, and *C. tertiotertiaria*^32^. The presence of this genus of beetles in both fossil assemblages and modern faunas allows us to make direct comparisons to modern analogues.

In this study we analyzed cuticle and organic preservation in Baltic amber insect inclusions using Synchrotron Micro-Computed Tomography (SR-$\upmu$CT) and Fourier Transform Infrared Spectroscopy (SR-FTIR), as well as Scanning Electron Microscopy (SEM) with Energy Dispersive Spectroscopy (EDS). Using these methods, we developed a sample selection process that can be used to justify more invasive analysis of amber inclusions. Comparisons with both extant and extinct beetle cuticle were used when applicable. The focus of the study is on a particularly well preserved specimen of *C. tertiotertiaria* from an amber sample selected for crack-out studies.

## Methods

### Sample preparation

The amber samples of interest were collected near the Sambian (Samland) Peninsula, Kaliningrad Region, Russia, and were commercially purchased. They reside as part of the Royal Saskatchewan Museum (RSM) collection as “RSKM_P3300.88”, which is referred to as “Baltic 83” herein, and “RSKM_P3300.144”, which is referred to as “Baltic 145” herein. Baltic 83 was chosen to undergo detailed analysis as it had a silvery appearance across the exoskeleton surface, showing that the exoskeleton separated from the surface of the amber. In this case it allowed analysis of cuticle structure via SEM. It also meant that cuticle chemical composition could be studied via FTIR, as flakes of cuticle could be removed easily.

The amber was cracked open along the middle of the insect body in order to perform the cuticle studies on some of the thickest cuticle sections in the body. This was accomplished by first polishing the amber until within 1 mm of the inclusion. A thin surface cut was made with a razor saw along the junction between the pronotum and elytra. The sample was then washed with de-ionized water to remove oils and other possible contaminants. Subsequently, the specimen was cracked by application of pressure along the cut with clean side-cutters, handled using surgical gloves above a sheet of aluminum foil. While the resultant split left a jagged edge, the use of this method ensured that we did not contact the inclusion surface directly, avoiding possible contamination. As a result, the sample was split into two pieces, one that covers the head and most of the thorax (Baltic 83A), and a larger piece that covers the entire abdomen (Baltic 83B). The two amber pieces were placed into centrifuge tubes for storage.

### Optical microscopy

All specimens were photographed using a Visionary Digital imaging system, which includes a Canon EQS 5D optical camera with a Canon MP-E 65 mm macro photography lens. Multiple photographs spanning a range of focal planes were taken in order to image an extended depth of field with high resolution. These images were processed in Helicon Focus 7.7.4 software, and further edited in Adobe Photoshop CS6 to produce figures ready for publication.

### Tomography

Micro-Computed Tomography with Phase Contrast Imaging ($\upmu$CT-PCI) scans were performed at the Biomedical Imaging and Therapy Facility (BMIT-ID) beam line of the Canadian Light Source synchrotron light facility. The beam energy used was 30 keV and the sample-to-detector distance was 0.1 m. 2500 projections over 180$^{\circ }$ were taken at an exposure time of 600 ms to produce the tomographic slices. The slices have a voxel resolution of 1.4 μm. Both pieces of Baltic 83 were imaged under similar parameters.

The free software Fiji (ImageJ) was used to prepare the CT slices (e.g. cropping, contrast enhancement, stacking of images). Dragonfly Pro (4.1) (https://www.theobjects.com/dragonfly/) software was used for segmentation of the CT model. The final 3D rendering of the sample was created using MeshLab for ambient occlusion and ZBrush for materials and shading. Blender (2.95) rendering software was used to stitch the two pieces of Baltic 83 back together into a single 3D model. The slice data and 3D models generated as part of the $\upmu$CT analysis in this study are available at MorphoSource (https://www.morphosource.org/projects/000495948).

### Infrared spectroscopy

For the FTIR analysis, small cuticle flakes that were loosely attached to the body cavity were extracted with a cleaned steel needle and moved to a centrifuge tube while working over an aluminum foil surface. For transfer to measurement slides at the synchrotron lab, the cuticle flakes were transferred to salt disks using the same needle technique.

FTIR scans were performed at the Mid-Infrared (Mid-IR) beam line of the Canadian Light Source synchrotron light facility. Infrared light is produced via bending magnet and sent through the Bruker Vertex 70v Interferometer. The Hyperion 3000 IR Microscope with 35× objective lens was used in order to focus the synchrotron beam onto the sample. For the samples of interest, the spectrometer operated in transmission mode, recording 256 sample spectra with a spot size of 12 μm and a spectral resolution of 4 cm$^{-1}$. For each scan, outlier spectra were removed from the set of 256 samples, and the remaining spectra were averaged to produce the raw FTIR spectra presented.

The infrared spectra were pre-processed (normalization, baseline correction, derivation, smoothing) with Quasar Spectroscopy (0.9)^35^, an open source addition to the Orange data analysis framework. The final FTIR and EDS spectra were visualized with the CERN ROOT data analysis framework (v6.20/06).

### Electron microscopy and spectroscopy

For the initial SEM analysis (before cuticle flake extraction), one of the two amber pieces of Baltic 83 was studied. Baltic 83B was chosen as it contained elytra exposed along its fractured surface. The piece was sputter coated in gold to improve the imaging (i.e., to minimize charging), while the other amber block was left untreated. The SEM analysis was performed with the JEOL JSM-6010LV microscope located at the Department of Earth and Atmospheric Sciences at the University of Alberta. Secondary Electron Imaging (SEI) mode was used to produce images with three-dimensional depth. Many images were taken of exposed cuticle cement layers on the crack-out surface. For comparison, cuticle layers of the elytra of a modern jewel beetle (Buprestidae: *Sternoceras ruficornis*) were also imaged.

Further SEM imaging and Energy Dispersive Spectroscopy (EDS) analysis (after cuticle flake extraction) were performed at the Faculty of Science’s electron microbeam facility, University of Regina, utilizing a Tescan Vega 3 microscope fitted with an EDAX-Apex EDS system. All samples were gold sputter coated at the Western College of Veterinary Medicine at the University of Saskatchewan. Secondary electron images were taken with a 10 KeV accelerating voltage. 200 s live times were used for the EDS area measurements in order to improve counting statistics. Nominal working distances were $\scriptstyle \sim$11–12 mm.

## Results

### Imaging

#### Optical microscopy

**Figure 1:**
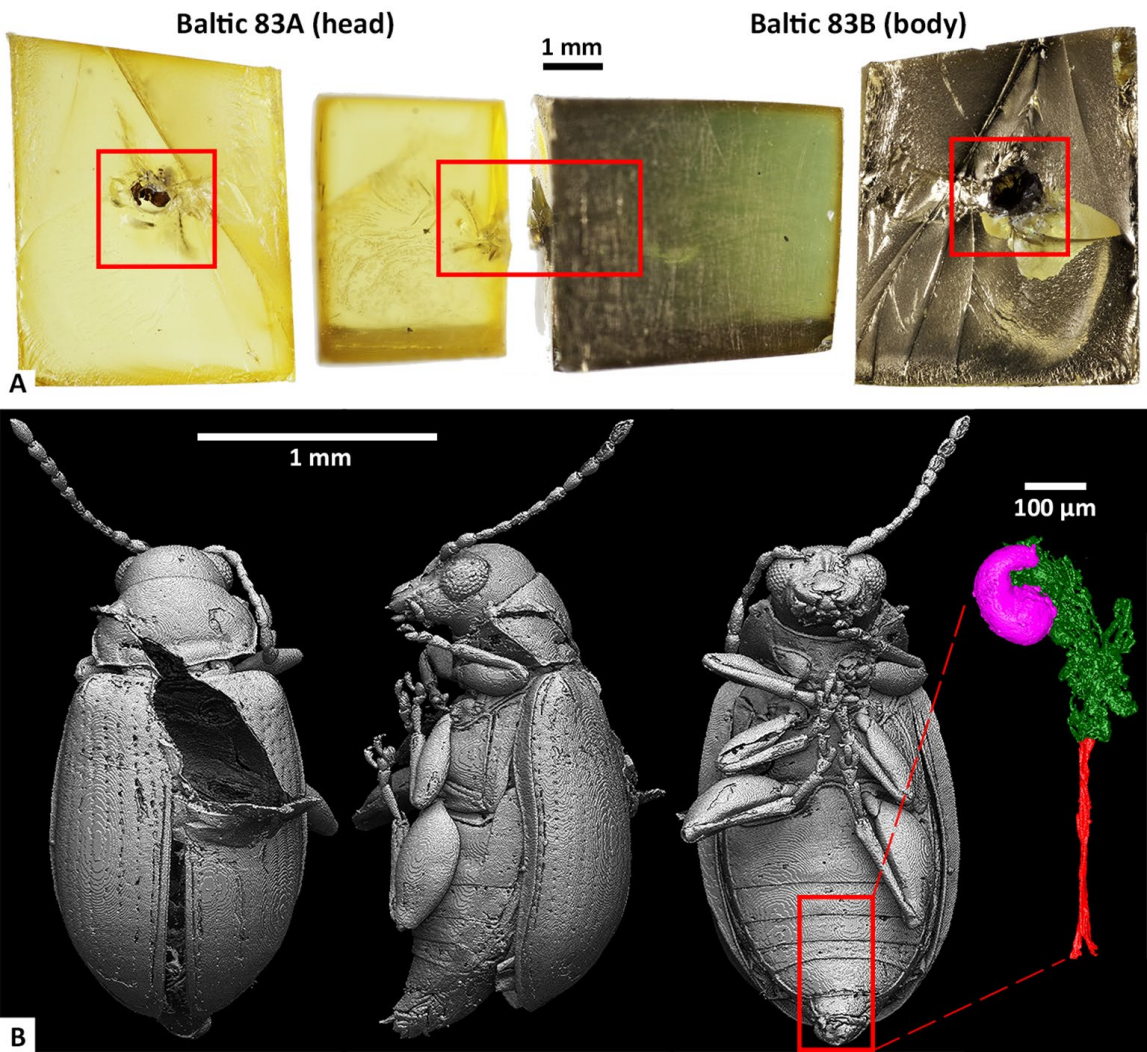
Photographs and 3D CT renders of Baltic 83. (**A**) Crack-out surface and side views of both pieces of Baltic 83, with location of the beetle inclusion marked. Baltic 83B was gold coated for SEM analysis. (**B**) CT renders of the dorsal, lateral and ventral views of the complete body of the *C. tertiotertiaria* specimen. Voxel size = 1.4 μm. The excavation on the dorsal side is indicative of the extraction location of the cuticle flakes for FTIR analysis. The excellent preservation allows the segmentation of the female genitalia of the insect on the right. Segmented out is the spermatheca capsule (pink), terminal portion (green), and spermathecal gland and duct (red). Genitalia labelling used from^36^.

Initial optical microscopy was performed to assess whether it was worth pursuing Baltic 83 for further imaging and organic spectroscopic studies. A comparison of excellent and poor preservation as seen in different Baltic amber insect inclusions can be made (Fig. S1), which we used as an initial selection criterion for organic spectroscopy. In both Baltic 83 and Baltic 145, exceptional preservation is suggested by the fact that the cuticle has pulled away from the surrounding amber, and original green reflective colour can be seen (Fig. S1A,B). SEM images (Fig. S1C) show clear layer preservation in the cuticle, which contrast with examples of common (but poor) level of preservation in Baltic amber inclusions via RSKM_P3300.83 (a flat bark beetle used for comparison). The cuticle of the insect is still attached to the amber wall and is shattered into many pieces (Fig. S1F). The cuticle appears as a single sheet in the SEM-SE image (Fig. S1G). Photographs of Baltic 83 were taken after the split into two amber pieces (Fig. 1A).

#### Tomography

High resolution CT scans of Baltic 83A and Baltic 83B were performed in order to resolve the features of the exterior and interior of the embedded insect and create a 3D model. The complete external CT render of Baltic 83 combines the separate amber scans (Fig. 1B). The dimensions of the sample are 2.2 mm × 1.0 mm × 0.8 mm. The apparent excavation on the dorsal side of the insect is where the cuticle flakes for the spectroscopic analysis were removed. The exterior cuticle preservation is well defined with clear elytral puncturing. The body cavity, while mostly empty, has remnants of soft tissues including preserved genitalia.

The excellent preservation of external and internal structures allowed a taxonomic assignment of the specimen. The following characteristics^32^ are used in determining the species as *C. tertiotertiaria* rather than other known fossil *Crepidodera*: longer prominent pronotal punctuation, distinctly smaller diameter of antennal socket, narrower antennal calli, and body size of just over 2 mm. The presence of the spermatheca capsule (shown in pink in the 3D rendering) within the genitalia indicates the insect is female.

#### Electron microscopy

**Figure 2:**
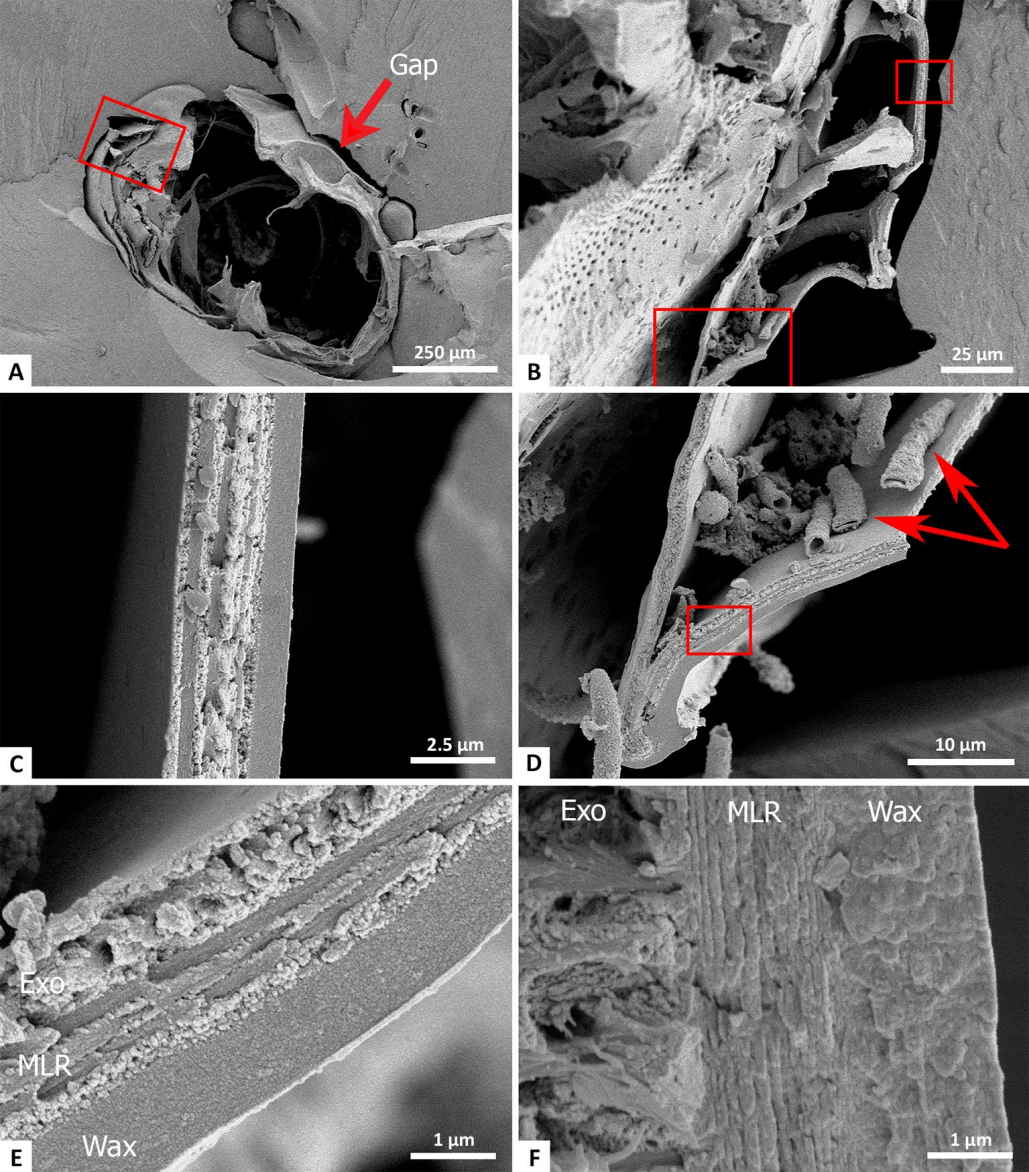
SEM secondary electron cuticle images of the exposed beetle in Baltic 83B taken prior to CT imaging and spectroscopy. (**A**) An overview picture of the exposed section. Exposed cuticle is seen on both the dorsal (bottom) and ventral (top) sides. (**B**) Magnification of a section of cuticle on the elytra. (**C**) Further magnification on the cuticle (top red box area in (**B**)). Preservation here is typical throughout the exposed cuticle. (**D**) A different cuticle section (bottom red box area in (**B**)) which features extensive trachea preservation (red arrows). (**E**) A close up of the exposed cuticle from the area of (**D**), featuring degraded multi-layer reflector (MLR) layering. (**F**) Comparison image of modern jewel beetle at the same scale as (**E**) for direct comparison of the MLR preservation. Exo: exocuticle, MLR: multi-layer reflector, Wax: waxy outer cuticle layer.

Electron microscopy allows imaging of the exposed cuticle of Baltic 83 in much higher resolution, allowing probing of individual cuticle layers. Initial SEM images of Baltic 83 (Fig. 2) were taken before the cuticle flakes were removed for spectroscopic work. Ventral and dorsal cuticle are exposed well and separated from the amber. The cuticle flakes for FTIR analysis were taken from areas where the cuticle plates were separated the farthest from the amber (Fig. 2A,B). This area was examined at higher magnification as well, illustrating microstructural preservation of the cuticle in this region (Fig. 2C,D), which is typical of the preserved cuticle structure of Baltic 83B. Thin-cuticled structures (such as trachea) and remnants of soft tissue are preserved within the body cavity near the cuticle sampling region (e.g., Fig. 2D), but they are also found in other areas of the exposed cuticle.

Degraded multi-layer reflector (MLR) structure can be seen in this region of the fossil cuticle, between the outer waxy layer and rest of the exocuticle (Fig. 2D,E). There appears to be a type of precipitate covering the layers partially, but remnants of lines can be seen. Comparison to fresh cuticle taken from a modern jewel beetle (Fig. 2F) supports this structural interpretation. Performing colour/reflectance analysis in a future study may be possible with these images. However, in most sections of the cuticle MLRs are not preserved (e.g., Fig. 2C). Due to this unexpected MLR preservation, this area of the cuticle was targeted for removal of flakes to look for organic signatures via spectroscopy.

### Spectroscopy

#### Infrared spectroscopy

**Figure 3:**
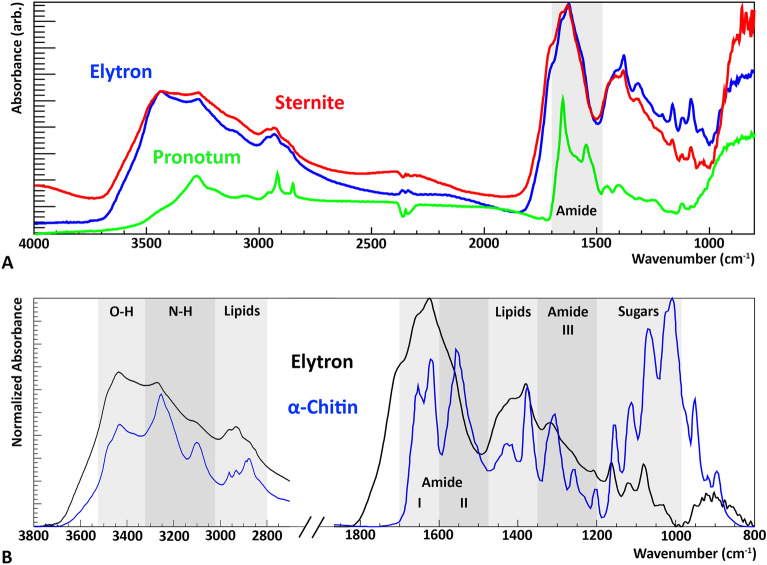
FTIR analysis of cuticle flakes from Baltic 83. (**A**) Averaged raw spectra for the three flakes analyzed, from the pronotum (green), sternite (red), elytron (blue). The pronotum flake was taken from Baltic 83A, while the other two were taken from Baltic 83B. General regions of amide/organic components are overlain for comparison. (**B**) High and low wavenumber regions comparing the normalized elytron spectrum to an $\upalpha$-chitin reference^40^. Normalization of the spectra for comparison was performed using the rubber band method over the region 3800-800 cm$^{-1}$ followed by scaling the largest peak to unity. Infrared band assignments for the elytron and $\upalpha$-chitin reference spectra can be found in Table 1.

Three flakes from the insect cuticle of Baltic 83 were chosen for infrared spectroscopy analysis: one from the pronotum from Baltic 83A, and others from the sternite and elytron from Baltic 83B (Figs. 3A, S2).

Amides are organic functional groups that make up the backbone of proteins, and are found in many organic molecules such as chitin, an amide derivative. Therefore, presence of amide bands in a sample is indicative of organic content. Characteristic amide vibrational modes include amide I ($\scriptstyle \sim$1650 cm$^{-1}$, C=O stretching), amide II ($\scriptstyle \sim$1550 cm$^{-1}$, N–H bending and C–N stretching), amide III ($\scriptstyle \sim$1400–1200 cm$^{-1}$, N–H bending and C–N stretching), and amide A and B ($\scriptstyle \sim$3300 cm$^{-1}$ and $\scriptstyle \sim$3070 cm$^{-1}$, N–H stretching)^37^. Characteristic “fingerprint” chitin peaks are sugar based and located in the low frequency region (1200–1000 cm$^{-1}$, C–O–C and C–O stretching). Main bands include^38^: $\scriptstyle \sim$1157 cm$^{-1}$, $\scriptstyle \sim$1113 cm$^{-1}$, $\scriptstyle \sim$1072 cm$^{-1}$, $\scriptstyle \sim$1021 cm$^{-1}$.

The pronotum spectrum features sharp bands known to be a part of organic/amide components. However, spectroscopic analysis of the pronotum spectrum reveals a strong protein signature that is likely due to human contamination (see Supplementary Information). The $\upalpha$-helix sub-band structure of the amide I protein (Fig. S3A; Table S1) is a signature that is found in human collagen: its presence in the pronotum sample likely refute the possibility of endogenous protein preservation, as beetle cuticle proteins are $\upbeta$-sheet dominated^39^. Consequently, Baltic 83A was not subjected to further analysis. Akin to the pronotum, the spectra for the sternite and elytron have notable bands in the organic/amide region (Fig. 3A). The sternite and elytron spectra appear to have the same signature, but since the elytron spectral peaks are better defined it will be the focus of the FTIR analysis herein (see Supplementary Information for sternite analysis; Table S2).

A spectral comparison can be made between the elytron spectrum and an $\upalpha$-chitin reference^40^ (Fig 3B; Table 1). The presence of bands in amide regions and C-H lipid signatures in the elytron spectrum support presence of organic content. Nearly all of the peaks of the elytron spectrum can be matched to peaks of the $\upalpha$-chitin reference. The peaks of the elytron spectrum are higher frequency by 3.7 ± 5.7 cm$^{-1}$ compared to the chitin reference. A second $\upalpha$-chitin reference has peaks that are 0.5 ± 5.7 cm$^{-1}$ lower than the elytron spectra^38^. Using FTIR, the main factor to delineate between the three polymorphs of chitin is the splitting of the amide I band. $\upgamma$ and $\upalpha$-chitin are known to have splitting of the Amide I band into two sub bands, with $\upbeta$-chitin being undivided^24^. Elytron bands at 1661 cm$^{-1}$ and 1623 cm$^{-1}$, tentatively assigned to amide I, match this splitting. Compared to the references of fresh chitin, the bands of the elytron spectrum have a generally broadened appearance. An experimental study on chitin degradation^41^ found overall loss of intensity (particularly in the characteristic sugar peaks) under exposure to high temperatures. This study also reported a new, large band at 1720 cm$^{-1}$ that was assigned as the carbonyl group C=O due to oxidation of the sample. The elytron band at 1713 cm$^{-1}$ was tentatively assigned to oxidation within the fossil. The reduced intensity of IR spectral bands compared to fresh chitin can be interpreted as the breaking of the chitin molecule into smaller fragment chains due to dehydration, deacetylation and depolymerisation reactions^27,41^. The molecular moieties identified in the elytron/sternite spectra are consistent with (at least partial) preservation of $\upalpha$-chitin.

Comparisons were made between several other possible organic contributions to the elytron and sternite cuticle flake FTIR spectra (Fig. S3): however, no potential sources of contamination were identified. Chitin-derived structures may undergo diagenesis that results in an aliphatic composition, known as kerogen^15^. Comparing the elytron spectrum to Type 1 kerogen spectra shows there may have been this type of alteration (Fig. S3C), but it is not extensive. Contributions from other organic sources appear to be negligible.

**Table 1:** Selected second derivative peaks of the elytron spectrum, with comparisons to two $\upalpha$-chitin references from shrimp.

Elytron peak frequency (cm$^{-1}$)	$\upalpha$-chitin Reference^40^ Peak frequency (cm$^{-1}$)	$\upalpha$-chitin Reference^38^ Peak frequency (cm$^{-1}$)	Band assignment	Tentative interpretation of organic components
3490	3483*	3479	$\nu$(O–H)	
3439	3428	3448	$\nu$(O–H)	
3266	3254	3268	$\nu _{as}$(N–H)	Amide A
3099	3100	3102	$\nu _s$(N–H)	Amide B
2967	2960	2965	$\nu _{as}$(CH$_3$)	Lipid
2927	2932	2927	$\nu _s$(CH$_2$)	Lipid
2873	2876	2883	$\nu _{as}$(CH$_3$)	Lipid
2850	–	–	$\nu _s$(CH$_2$)	Lipid
1772	–	–	?	
1713	–	–	$\nu$(C=O)	Oxidation
1661	1652	1660	$\nu$(C=O)	Amide I
1623	1621	1627	$\nu$(C=O)	Amide I
1558	1556	1558	$\nu$(C–N) + $\delta$(N–H)	Amide II
1453	–	–	$\delta$(CH$_2$)	Lipid
1418	1428	1422	$\delta$(CH$_2$)	Lipid
1379	1376	1376	$\delta$(CH) + $\delta$(C–CH$_3$)	Lipid
1315	1308	1312	$\nu$(C–N) + $\delta$(N–H)	Amide III
1259	1260	1255	$\delta$(N–H)	Amide III
1207	1207	–	$\delta$(N–H)	Amide III
1163	1156	1157	$\nu _{as}$(C–O–C, ring)	
1120	1114	1113	$\nu$(C–O)	Sugar
1080	1069	1072	$\nu$(C–O)	Sugar
1032	1029*	–	$\nu$(C–O)	Sugar
1015	1008	1021	$\nu$(C–O)	Sugar
992	995*	–	?	
955	952	957	$\gamma$(CH$_3$)	

Band assignments are indicative of components identified by^38^. Frequency bands labeled with “*” are missing from the articles data table, but are found from our own analysis of the presented spectra.

#### Energy dispersive spectroscopy

**Figure 4:**
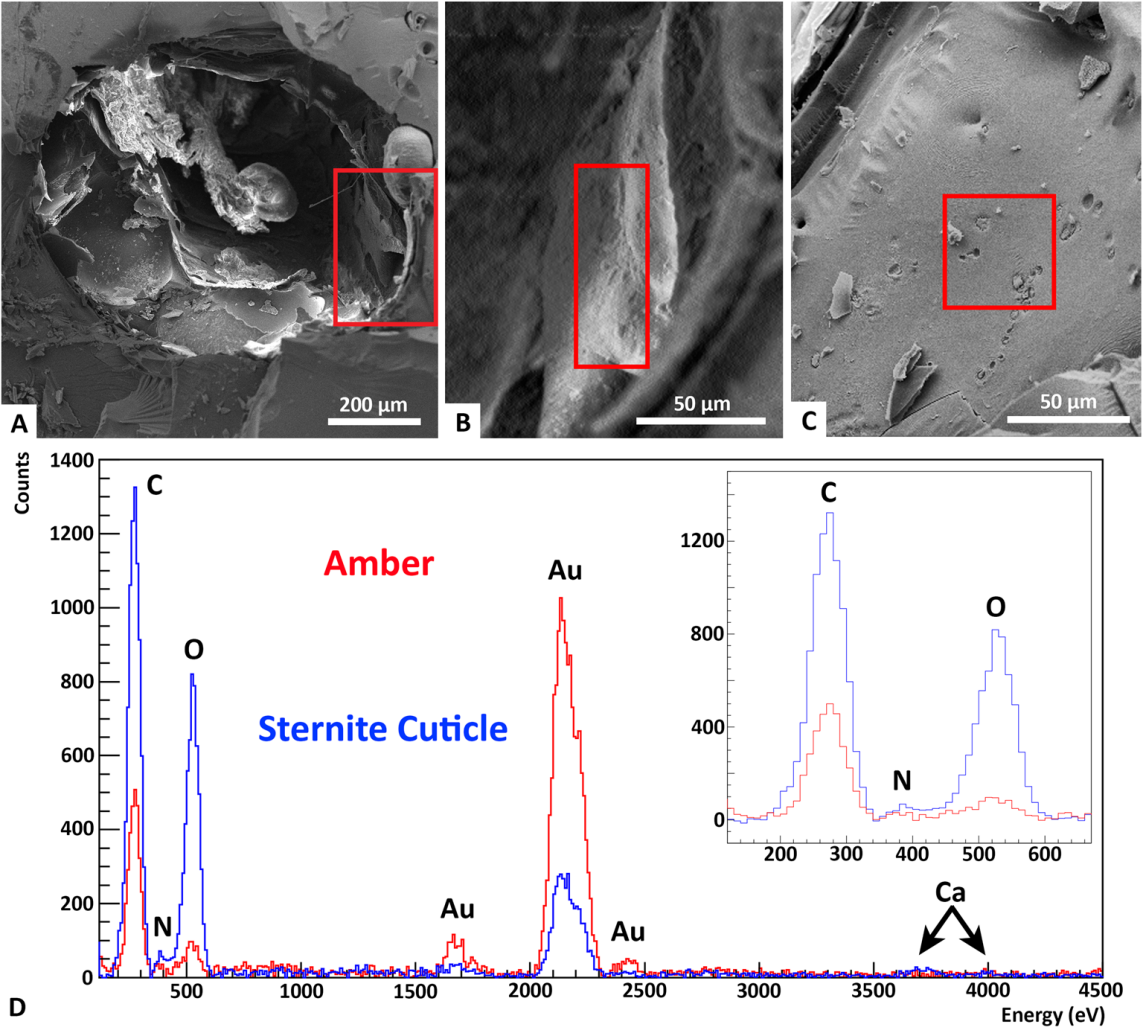
SEM-EDS analysis of Baltic 83. (**A**) SE image of exposed beetle in Baltic 83B after the removal of flakes for FTIR analysis. (**B**) Section of exposed sternite cuticle with marked location for EDS probing. (**C**) Section of nearby amber with EDS probing location. (**D**) EDS spectral comparison of the sternite cuticle (blue) and Baltic amber (red), with inset of a magnification of the low eV region. Gold M peaks between 1500 and 2500 eV are due to the coating process and are not native to the sample. The amber only contains carbon and oxygen, while the cuticle also contains trace amounts of nitrogen and calcium.

Combining SEM images with EDS allows us to interpret the chemical composition of the cuticle preserved in Baltic 83B in situ (Fig. 4). EDS results show that the sternite cuticle is composed almost entirely of elements associated with organics: carbon (K$\upalpha$ 277 eV), oxygen (K$\upalpha$ 525 eV), and trace amounts of nitrogen (K$\upalpha$ 392 eV). The cuticle spectrum also has measurable quantities of calcium (K$\upalpha$ 3690 eV and K$\upbeta$ 4013 eV). No other elemental peaks are present past 4500 eV. As amber provides a relatively closed system it is unlikely that the calcium detected (Fig. 4D) is from an external source outside the insect, such as calcium carbonate crystals. Calcium, as well as other metals, are known to act as nuclei for cross-linking between $\upalpha$-chitin and proteins in the cuticle, in the process of sclerotization^38^. Elytron cuticle was also probed and has similar composition (Fig. S4). Quantitative analysis on the spectra were performed (including calculation of carbon-oxygen ratios); however, we cannot find evidence for chitin preservation from the SEM-EDS results alone due to the uncertainty in EDS quantitative analysis (see Supplementary Information; Tables S3, S4). Quantitative analysis does suggest that proteinaceous contribution in the elytron/sternite cuticle is minimal due to the low relative amounts of nitrogen and oxygen.

## Discussion

Initial findings of excellent preservation in Baltic 83 can be discerned with SEM and $\upmu$CT imaging. The specimen features preserved structural colour with degraded multi-layer reflectors in the cuticle, as well as internal soft tissues such as genitalia. Fossil structures that are preserved well morphologically give motivation for further analysis using spectroscopy in search of organics. The balance of evidence suggests vestigial cuticle chitin preservation in Baltic 83B from the results of the FTIR analysis. Almost all of the elytron and sternite flake spectral IR peaks can be matched using an $\upalpha$-chitin standard. The chitin moieties appear to be reduced in intensity across the entire spectra, with appearance most comparable to thermally degraded chitin^41^. The SEM-EDS results provide the qualitative chemical composition of the preserved cuticle, showing its composition is dominated by carbon and oxygen, with trace amounts of nitrogen and calcium.

With any claim of organic preservation in fossils, caution must be taken on possible forms of contamination. Results of the pronotum FTIR spectrum show that it is most likely dominated by a human contaminant, which makes the spectral results void. There may be underlying endogenous organic material in the exposed cuticle of Baltic 83A, but it would be ambitious to distinguish this material from overlapping of a strong exogenous protein signal using the spectral methods of the study. Baltic 83A was not subject to the initial SEM analysis and thus was handled less than Baltic 83B, demonstrating how easily contamination can happen even while actively controlling for it (as described in the methods). Naturally, a concern may be raised about a potential chitin contaminant in Baltic 83B from the sternite and elytron samples, akin to Baltic 83A. However, this is unlikely because chitin is not a possible contaminant from humans. There is also a small possibility of fungal contamination contributing to an $\upalpha$-chitin signature, but there is no evidence of this from the SEM images of the cuticle. Fungi cells are commonly 2 to 10 μm in diameter^42^ and thus would be expected to be observed in the SEM analysis (Fig. 2C–E).

The FTIR spectra presented in this study appears visually different compared to other identified fossil chitin signatures from shale. In a 200 Ma gastropod egg^28^, there appears to be very little alteration to the shape and position of the spectral chitin peaks and can therefore be matched unequivocally to a $\upbeta$-chitin reference. Most reported fossil chitin FTIR spectra (34 Ma cuttlebone^27^, 505 Ma sponge^30^, 810–715 Ma fungi^31^) appear more similar to standard protein spectra rather than solely chitin. Distinctly separated amides I and II are yielded, but with a low frequency region (<1500 cm$^{-1}$) that is unrecognizable compared to the presented chitin references with many missing characteristic sugar peaks. From the FTIR analysis, the quality of the spectrum in our studies seem to show stronger evidence for $\upalpha$-chitin than previously attempted analyses reported in the literature. Beyond FTIR and EDS analyses, previous works have based their fossil chitin claims on other methods including immunohistochemistry, chromatography, Raman spectroscopy, mass spectroscopy, X-ray absorption near edge structure, and scanning transmission X-ray microscopy^27–31^. We plan to provide a more thorough multi-technique quantitative analysis of the organics present in the cuticle of Baltic 83 and other equally well-preserved Baltic amber inclusions in the RSM collection in a future study.

Generally, components of the original chitin-protein complex tend to transform into complex geopolymers in fossilized insect cuticle^43,44^. However, spectral results in this study suggest organics left in the cuticle of Baltic 83 consist predominately of degraded endogenous chitin. Contribution due to protein and other possible derived diagenetic components are minimal, hinting at an unexplored taphonomic pathway for the beetle cuticle. We propose that amber’s protective properties, along with separation of the insect cuticle from the interior amber wall as the main explanation for the high quality of cuticle preservation observed. If separation occurred before the resin fully polymerised, it would limit the interaction of cuticle with acids, alchohols and trace quantities of other compounds that are present alongside the main framework of terpenoids in the resin^45^ surrounding the amber inclusion. This separation of the cuticle from the amber is thought to be the result of especially rapid drying of the beetle in the amber that occurred after initial flows of resin^46^, limiting opportunities for hydrolysis among the original materials of the amber inclusions^14^. Furthermore, the $\upalpha$-chitin found in insect cuticles is more thermally stable than $\upbeta$-chitin. Beetles are known to have highly cross-linked thick sclerotized cuticles which increases their resistance to degradation. As a result, they survive more commonly in the fossil record than other arthropods^30^.

In the 1990s, crack-out studies performed on insects in amber identified remnants of tissues from muscles, nervous systems, and digestive systems (e.g.,^47^), and some even claimed the preservation of DNA^48^. Since this time, crack-out studies of organic remains inside amber inclusions seem to have dwindled, with this form of research attracting less interest. Recent crack-out studies^49^ have recovered heavily degraded DNA from 60-year-old resin, but ultimately did not recommend applying this to amber samples. Another study^8^ found preserved amino acids from feathers found in Baltic and Burmese amber via crushing of the amber, providing promise for use of destructive analysis for biomolecular research in deep time. However, the majority of related soft tissue studies that have aimed to sample insects or other inclusions in situ within amber have done so directly and without cracking open specimens—instead these projects have involved polishing the amber piece to within a millimetre of the inclusions sample surface, mainly to probe for pigments^13^ or structural colours^4^.

The absence of modern crack-out studies is likely due to: (1) the risk of permanently damaging high quality specimens that may be in short supply; (2) our inability to predict the extent of soft tissue preservation before cracking specimens open to examine their tissues; (3) the need for advanced analytical equipment and multidisciplinary teams in order to fully characterize organic remains; and (4) the problems associated with ancient DNA and protein preservation claims of the 1990’s, which were subsequently classified as insufficiently proven, contamination, or non-reproducible^50^.

The quality of amber preservation observed here appears to be widespread among Baltic and Dominican amber specimens that have been subjected to CT scans to date (reviewed recently by^51^), and it may even extend back to the Cretaceous deposits. Recent work on colour preservation in Burmese amber^4^ has revealed similar structural preservation in cuticle samples from 35 individuals across three insect orders. Extensive soft tissue preservation has also been documented as far back as the Cretaceous^51^, with much of the musculature and digestive system preserved in original position. Without crack-out studies, we still have a very limited understanding of what these preserved structures are composed of at a chemical or molecular level. With CT scanning becoming more prevalent as a research technique, it is possible to identify and target tissues within an increasing range of specimens. The recent growth in museum amber collections also means that specimens of common taxa are increasingly available for crack-out studies. Also, access to synchrotron light sources for CT scanning and spectroscopy techniques like FTIR improves signal-to-noise ratio and acquisition times^15^, allowing quick screening for exceptional preservation of many samples. While the preservation of decay-prone molecules such as DNA or proteins is still debated (see^52^ for a recent review), more recalcitrant organic macromolecules such as carbohydrates or lipids may be more relevant for recovery as fossilized organic material on geological timescales, and amber may be an ideal medium for the preservation of this material.

Conventional views held in paleontology, as in any science, should always be challenged. Scanned specimens are no longer lost to science if destructive sampling is conducted carefully, and destructive sampling allows a more rigorous analysis of preserved material. Crack-out studies are still a valuable source of information, and they should be considered as a supplement to some of the latest techniques, particularly when dealing with inclusions of common fossil taxa. Finding remnant organic material such as chitin earlier in the geological record will help redefine taphonomic limits and provide a more life-like characterization of ancient ecosystems. The results of this study give motivation to further investigate traces of organic material in fossils, particularly when they are preserved in amber.

## Supplementary Information

Supplementary Information.

## Data Availability

Data is available upon reasonable request to the corresponding author.
