# Supplementary material for: Morphological and organic spectroscopic studies of a 44-million-year-old leaf beetle (Coleoptera: Chrysomelidae) in amber with endogenous remains of chitin
Source: Sci Rep. 2023 Apr 11;13:5876. doi: 10.1038/s41598-023-32557-w (PMC10090159; doi:10.1038/s41598-023-32557-w)
Supplement: Supplementary file 1 — Supplementary Information. [file 41598_2023_32557_MOESM1_ESM.pdf]

Supplementary Information

Morphological and organic spectroscopic studies of a 44-million-year-old leaf beetle (Coleoptera: Chrysomelidae) in amber with endogenous remains of chitin

Jerit L. Mitchell[1,*], Ryan C. McKellar[2,3,4], Mauricio Barbi[1], Ian M. Coulson[5], and Andris Bukejs[6]
[1]Department of Physics, University of Regina, Regina, SK, S4S 0A2, Canada.
[2]Royal Saskatchewan Museum, 2445 Albert St., Regina, SK, S4P 4W7, Canada.
[3]Department of Biology, University of Regina, Regina, SK, S4S 0A2, Canada.
[4]Department of Ecology & Evolutionary Biology, University of Kansas, Lawrence, Kansas, 66045, USA.
[5]Department of Geology, University of Regina, Regina, Saskatchewan, S4S 0A2, Canada.
[6]Institute of Life Sciences and Technologies, Daugavpils University, Vienîbas 13, Daugavpils, LV-5401, Latvia.
*Corresponding Author: jlm696@uregina.ca

Supplementary Methods and Results

FTIR

2nd Derivative Analysis

2nd derivative analysis can be used to determine the precise positions of peaks, and deconvolute the sub-bands of the fingerprint region of the mid infrared spectrum. An 11-point Savitzky–Golay filter was applied to the raw cuticle flake spectra. The three flake spectra can be overlain with their corresponding 2nd derivative spectrum (Fig. S2). Tables S1 and S2 list the 2nd derivative peaks for the pronotum and sternite sections, tentative band assignments, as well as comparison to human protein and α-chitin references, respectively.

Reference Comparison

Possible organic contributions/contaminants for the cuticle flake spectra were examined in detail (Fig. S3). Inorganic contributions are expected to be minimal for amber inclusions. There are three protein references: Bovine Serum Albumin[1] (BSA, a standard protein reference), human collagen[2], and extant insect cuticle protein[3]. Other non-proteinaceous references include Baltic amber[4], α-chitin[5] (the other main component of cuticle), and Type 1 kerogen[6] (an aliphatic structure that can be derived from diagenesis of chitin). Normalization of the spectra for comparison was performed using the rubber band method over the entire spectral region. Protein references were compared by normalizing the amide I peak to unity. The other references had their largest peak normalized to unity.

The pronotum spectrum (Fig. S3 A) resembles the BSA spectrum closely, with distinct peaks in amide regions. The human collagen spectrum also matches very well. The pronotum spectrum is consequently dominated by protein. The extant insect cuticle protein contains the same main amide I and II peaks, but they are redshifted by about ~20 cm^{-1} compared to the mammal proteins. This is because the β-sheet secondary structure dominates the amide peaks in insect cuticle protein[3]. The small redshift of the amide I α-helix band on the pronotum spectrum with

respect to the BSA and human collagen spectra (1656 cm^{-1}) could be explained by the presence of a broad peak at 1593 cm^{-1} that is not accounted for by the protein references. The amide III shoulder at 1236 cm^{-1} is most similar to the BSA spectrum rather than the human collagen or insect cuticle protein. The main Baltic amber peaks are not represented in the pronotum spectrum. The largest Baltic amber peaks are outside the frequency range of the bands in the amide I range, even though the other main peak is located at the exact position of the first lipid peak (1451 cm^{-1}). The kerogen spectrum features a broad peak at 1450 cm^{-1}, but the absence of the other peaks means there is likely no contribution to the pronotum spectrum. The α-chitin spectrum features numerous sharp peaks, including ones located on the amide I and amide II α-helix bands, although the non-appearance of the low frequency chitin bands (1500-1000 cm^{-1}) makes significant contribution unlikely. As a result, we conclude that it is likely that the organics found in the pronotum FTIR spectrum are due to human protein contamination rather than beetle protein or other organics. We suggest that piece Baltic 83A in which the pronotum flake had been extracted has been contaminated at some point during the sample preparation process.

The elytron and sternite spectra (Fig. S3 B, C) are very similar and will be discussed in the same context. Amide I and II bands (1659, 1549 cm^{-1}) and peaks at 1454 cm^{-1} and 1080 cm^{-1} of human protein are matched in the elytron spectrum. However, peaks at 1743 cm^{-1}, 1396 cm^{-1}, and the amide III peak (1238 cm^{-1}) are not represented. Therefore, the chance of human protein contamination is low in the elytron/sternite spectra. The absence of the lower frequency amide II peak (1512 cm^{-1}) found from the extant beetle cuticle protein suggests endogenous protein contribution is minimal in the elytron/sternite. No contribution due to Baltic amber is evident; peaks at 1450 cm^{-1}, 1380 cm^{-1}, and 1160 cm^{-1} are present but the absence of the largest peak at 1738 cm^{-1} discredits amber contribution. Type I kerogen peaks at around 1374 cm^{-1}, 1450 cm^{-1} are seen in the spectra, but the small band at 1695 cm^{-1} and large broad band at 1605 cm^{-1} are not. Kerogen contribution is therefore unlikely. As discussed in the manuscript, α-chitin references account for almost all of the peaks seen in the spectra and thus are sufficient for describing the bulk of the organic moieties found in the elytron and sternite spectra.

SEM-EDS

Quantitative Analysis

Since chitin has the chemical formula $C_8H_{13}O_5N$, it is expected to have a carbon-to-oxygen ratio of C/O = 1.6 in elemental analysis. From the quantitative analysis (Table S3, S4), the calculated atomic carbon-to-oxygen ratio is C/O = 1.44 and C/O = 1.49 for the sternite and elytron cuticle, respectively, close to the theoretical ratio. For the amber spectrum, the calculated value is C/O = 6.33, clearly distinguished from the cuticle. Due to the high uncertainty in the statistics, no claim of chitin preservation can be made from the EDS analysis. We can find support, however, for the idea that very little if any proteins have survived in the original chitin-protein matrix of the cuticle. For example, a low nitrogen content (~7%) is indicative of this[7], and as well the C/O value would be much lower in a protein containing matrix[8]. These features also make possible modern or human protein contamination unlikely.

References

[1] Sukumaran, S. Protein secondary structure elucidation using FTIR spectroscopy (Application Note No. AN52985). Retrieved from Thermo Fisher website: AN52985-protein-secondary-structure-elucidation-using-ftir-spectroscopy.pdf (thermofisher.com) (2022).

[2] Sandt, C. Liver Spectroscopy (Collagen) [Data Set]. *Quasar 0.9* (2017).

[3] Iconomidou, V. A., Chryssikos, G. D., Gionis, V., Willis, J. H. & Hamodrakas, S. J. "Soft"-cuticle protein secondary structure as revealed by FT-Raman, ATR FT-IR and CD spectroscopy. *Insect Biochem. Mol. Biol.* **31**, 877–885, DOI: 10.1016/S0965-1748(01)00033-9 (2001).

[4] Causey, F. Ancient Carved Ambers in the J. Paul Getty Museum. *Getty Publications* (2020).

[5] Hassainia, A., Satha, H. & Boufi, S. Chitin from *Agaricus bisporus*: Extraction and characterization. *Int. J. Biol. Macromol.* **117**, 1334–1342, DOI: 10.1016/j.ijbiomac.2017.11.172 (2018).

[6] Mroczkowska-Szerszé n, M. & Jankowski, L. S. The organic matter type in the shale rock samples assessed by FTIR-ATR analyses fossil resins view project application of spectroscopic techniques in several aspects of rock analysis view project. NAFTA-GAZ **6**, 361–369, DOI: 10.17632/rx8jp7chkv.2 (2015).

[7] Cárdenas, G., Cabrera, G., Taboada, E. & Miranda, S. P. Chitin characterization by SEM, FTIR, XRD, and 13C cross polarization/mass angle spinning NMR. *J. Appl. Polym. Sci*. **93**, 1876–1885, DOI: 10.1002/app.20647 (2004).

[8] Godigamuwa, K., Nakashima, K., Tsujitani, S. & Kawasaki, S. Fabrication of silica on chitin in ambient conditions using silicatein fused with a chitin-binding domain. *Bioprocess Biosyst. Eng.* **44**, 1883–1890, DOI: 10.1007/s00449-021-02568-w (2021).

[9] Bobroff, V., Chen, H. H., Javerzat, S. & Petibois, C. What can infrared spectroscopy do for characterizing organic remnant in fossils? *TrAC - Trends Anal. Chem.* **82**, 443–456, DOI: 10.1016/j.trac.2016.07.005 (2016).

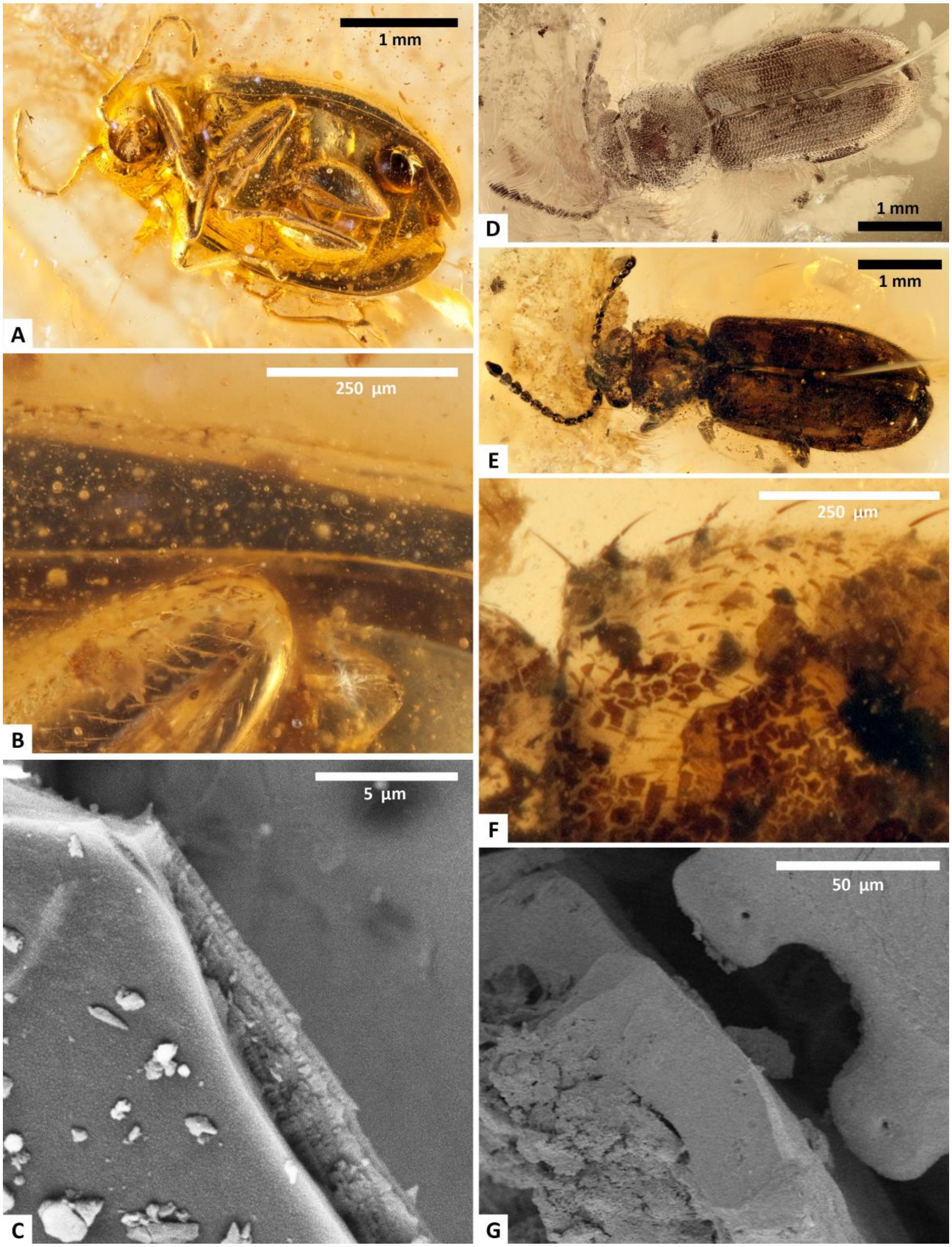

Figure S1: (A,B,C) (left column): RSKM_P3300.144 (Baltic 145), an example of excellent cuticle preservation in Baltic amber. Figures (D,E,F,G) (right column): RSKM_P3300.83, an example of poor cuticle preservation in Baltic amber. (A,D,E): overview images of the insects. (B,F): close up of the upper thorax of the insects. (C,G): SEM SE images of the cuticle.

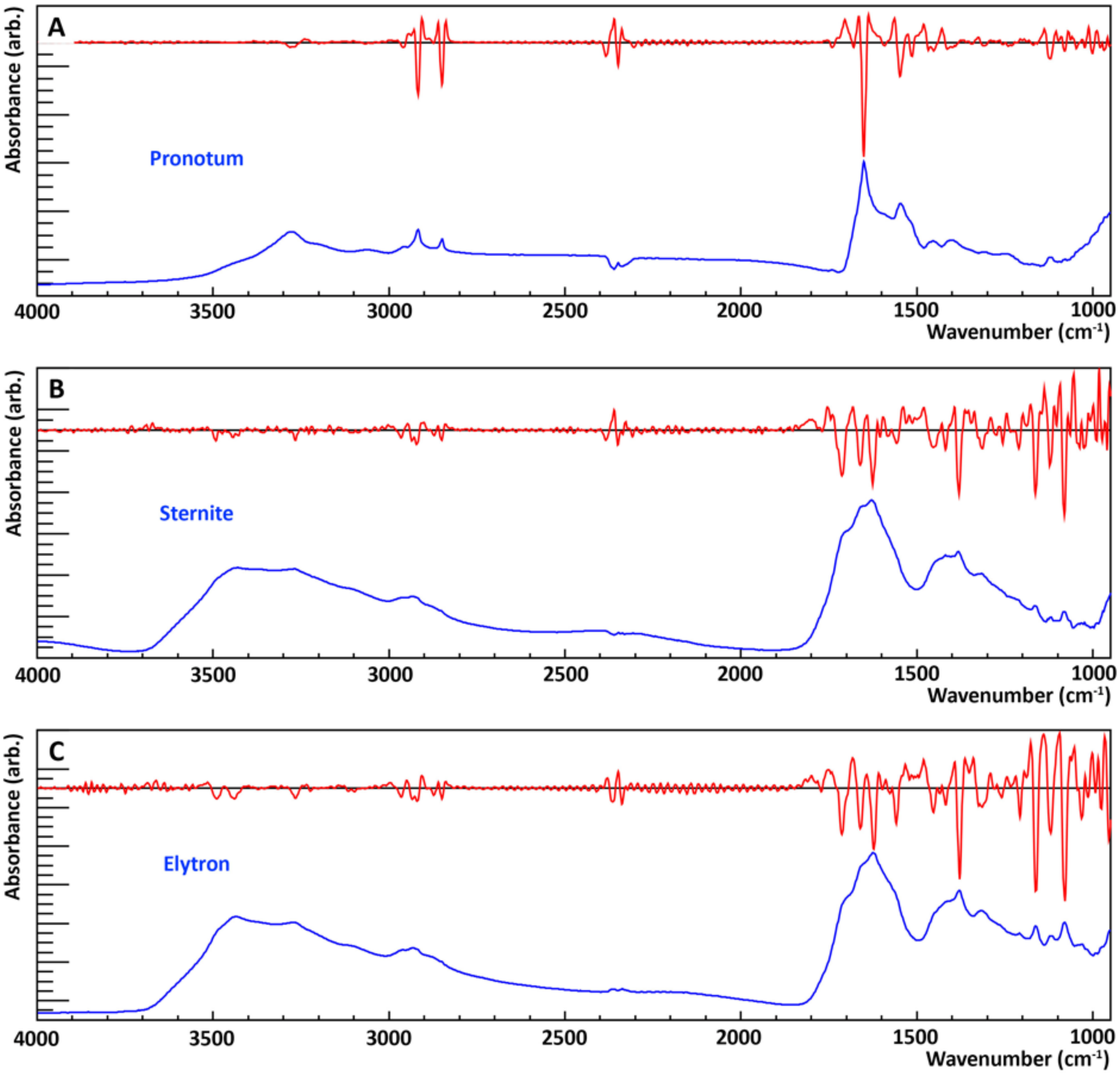

Figure S2: Pronotum (A), sternite (B) and elytron (C) flake spectrum with overlayed 2nd derivative. The 2nd derivative spectrum is produced from an 11-point Savitzky–Golay Filter and scaled to fit into the graphing window.

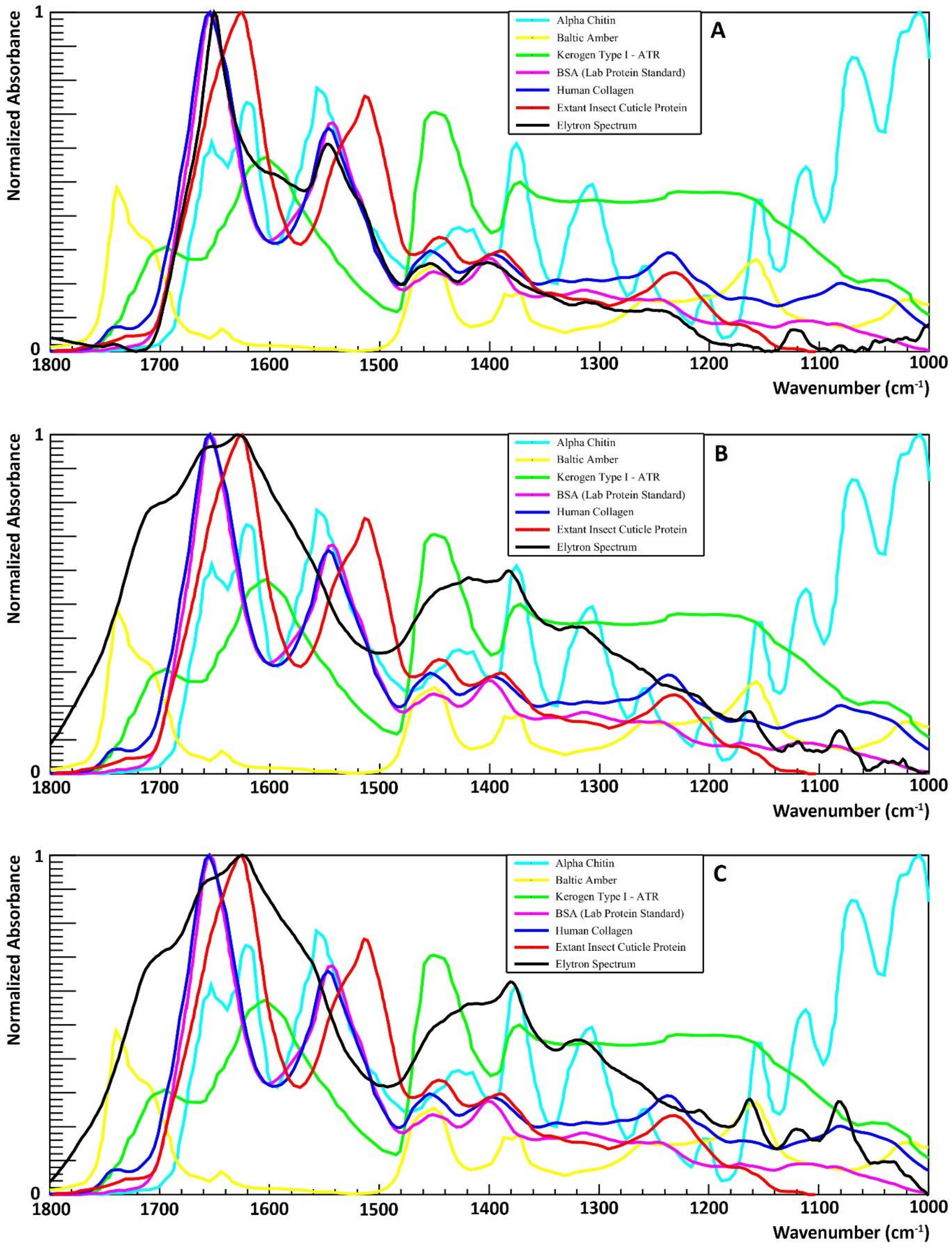

Figure S3: Low wavenumber region reference comparison – cuticle flake spectra superimposed on spectra of potential organic contributions. (A) Pronotum (B) Sternite (C) Elytron. All spectra are normalized to their largest peak over the entire mid IR spectrum.

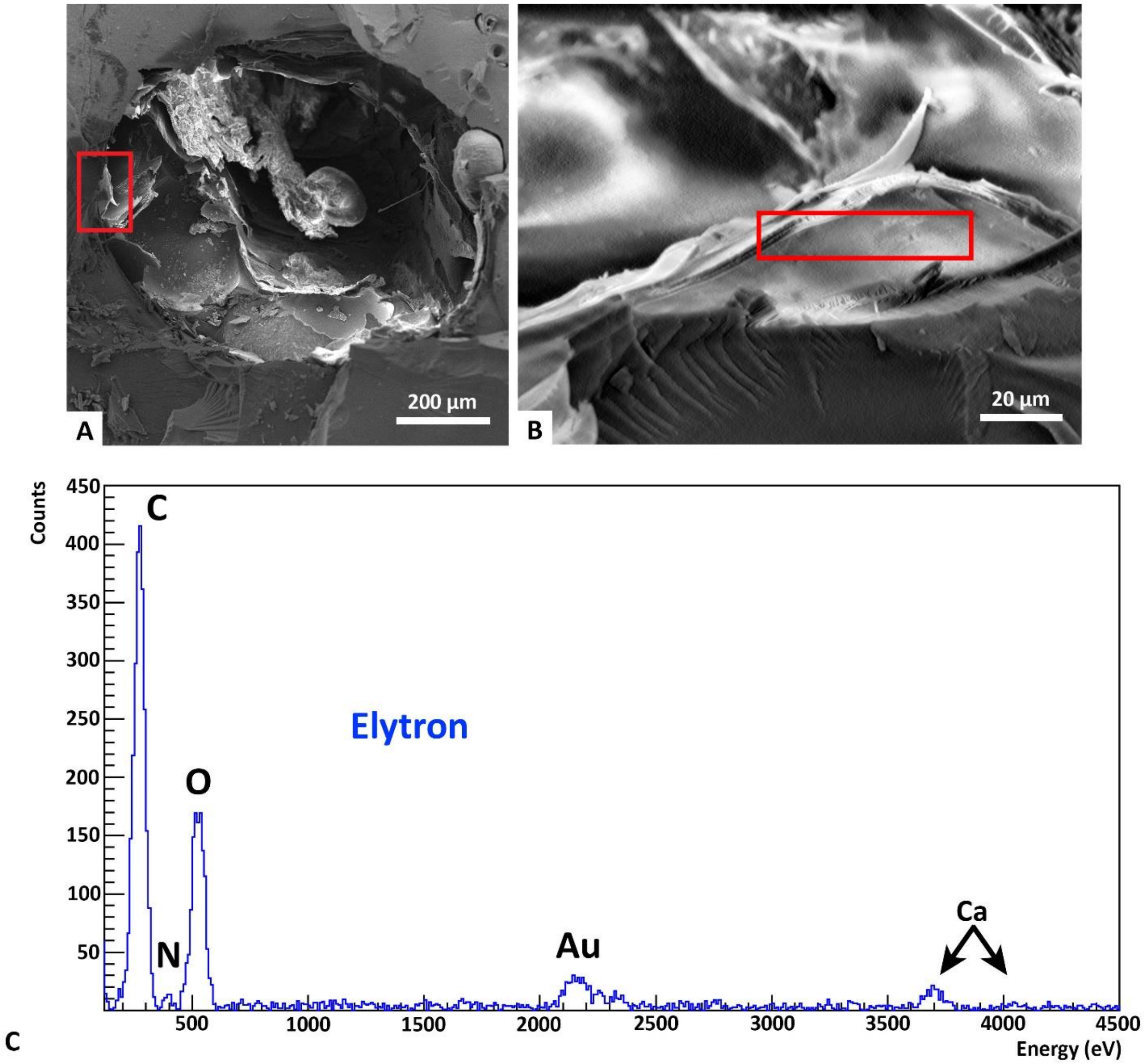

Figure S4: SEM-EDS analysis of Baltic 83B. (A) SE image of crack-out section of Baltic 83B, after removal of flakes for FTIR analysis. (B) Piece of exposed elytron cuticle with location for EDS probing. (C) EDS spectrum of the elytron cuticle. The cuticle contains carbon, oxygen and trace amounts of nitrogen and calcium.

Frequency (cm^{-1})	Protein Reference (Human Collagen[2]) (cm^{-1})	Band Assignment	Tentative Interpretation of Organic Components
3278		ν(N-H)	Amide A
3061		ν(N-H)	Amide B
2918		$\nu_{as}(CH_2)$	Lipid
2850		$\nu_{s}(CH_2)$	Lipid
1741	1743	ν(C=O)	Acid
1651	1659	ν(C=O)	Amide I
1593	-	ν(C=O)	?
1547	1549	ν(C-N), δ(N-H)	Amide II
1460	1454	$\delta_{as}(CH_2)$	Lipid
1405	1396	$\delta_{s}(CH_2)$	Lipid
1236	1238	ν(C-N), δ(N-H)	Amide III
1122		?	?
1081	1080	ν(C-O)	Sugar

Table S1: Selected main peaks of the pronotum spectrum, with comparison to a human collagen reference. Band assignments are indicative of components identified by [9].

Sternite Peak Frequency (cm^{-1})	α-chitin Reference[5] Peak Frequency (cm^{-1})	α-chitin Reference[7] Peak Frequency (cm^{-1})	Band Assignment	Tentative Interpretation of Organic Components
3492	3483	3479	ν(O-H)	
-	3428	3448	ν(O-H)	
3267	3254	3268	ν_{as}(N-H)	Amide A
3099	3100	3102	ν_{s}(N-H)	Amide B
2966	2960	2965	$\nu_{as}(CH_3)$	Lipid
2928	2932	2927	$\nu_{s}(CH_2)$	Lipid
2872	2876	2883	$\nu_{as}(CH_3)$	Lipid
2851	-	-	$\nu_{s}(CH_2)$	Lipid
-	-	-	?	
1713	-	-	ν(C=0)	Oxidation
1661	1652	1660	ν(C=0)	Amide I
1625	1621	1627	ν(C=0)	Amide I
1558	1556	1558	ν(C-N) + 8(N-H)	Amide II
1453	-	-	$\delta(CH_2)$	Lipid
1419	1428	1422	$\delta(CH_2)$	Lipid
1381	1376	1376	δ(CH) + $\delta(C\text{-}CH_3)$	Lipid
1315	1308	1312	ν(C-N) + δ(N-H)	Amide III
1256	1260	1255	δ(N-H)	Amide III
1211	1207	-	δ(N-H)	Amide III
1164	1156	1157	ν_{as}(C-O-C, ring)	
1122	1114	1113	ν(C-O)	Sugar
1081	1069	1072	ν(C-O)	Sugar
-	1029*	-	ν(C-O)	Sugar

-	1008	1021	ν(C-O)	Sugar
-	995*	-	?	
-	952	957	γ(CH_3)	

Table S2: Selected peaks of the sternite spectrum, with comparisons to two α-chitin references from shrimp. Band assignments are indicative of components identified by [7]. Frequency bands labeled with "*" are missing from the articles data table, but are found from our own analysis of the given spectra.

	Element	Weight %	Atomic %	Error %
Amber	C	82.61	86.36	43.91
	O	17.39	13.64	69.57
Sternite	C	44.93	52.83	9.61
	N	8.80	8.87	24.76
	O	41.54	36.64	13.26
	Ca	4.74	1.67	25.30

Table S3: EDS statistics table for the amber and sternite section.

	Element	Weight %	Atomic %	Error %
Elytron	C	42.34	53.14	8.45
	N	5.41	5.81	39.30
	O	37.84	35.62	13.96
	Ca	14.41	5.43	14.41

Table S4: EDS statistics table for an elytron section.
